# Methods for estimating beneficiary populations targeted by health and nutrition interventions for women, pregnant women, infants, and young children

**DOI:** 10.1093/aje/kwae469

**Published:** 2024-12-27

**Authors:** Soyra Gune, Phuong H Nguyen, Suman Chakrabarti

**Affiliations:** International Food Policy Research Institute, New Delhi, India; International Food Policy Research Institute, Washington DC 2005, United States; International Food Policy Research Institute, Washington DC 2005, United States

**Keywords:** health and nutrition interventions, maternal and child health, methods, population

## Abstract

Utilization of maternal and child interventions is typically tracked in low- and middle-income countries (LMICs) using coverage estimates from population representative surveys. These estimates cannot be directly applied to assess resource gaps in intervention delivery for which data on the population eligible are required. Moreover, coverage improvements may not necessarily reflect an expansion in utilization because of a decline in the population eligible. We develop a method to estimate the populations eligible for interventions across the continuum of care. The method uses data from the World Population Prospects and the Demographic Health Survey, data sources that are available for most LMICs. Additionally, we develop a method to estimate the eligible population covered by each intervention. Using the illustration of India, we estimate populations eligible for and covered by interventions during preconception, pregnancy, delivery, lactation, and childhood. We find that between 2015 and 2020, the eligible population declined for all beneficiary groups. Additionally, coverage expansion was not entirely driven by an increase in the population accessing an intervention, but rather also by a decline in the eligible population. Our illustration highlights the importance of including population estimates alongside coverage for interventions, particularly in LMIC contexts due to changing fertility dynamics.

## Introduction

Achieving universal health coverage and reducing child malnutrition and mortality have been recognized as Sustainable Development Goals.^1^ Within the broader objective of universal health coverage, focus has been given to the first 1000 days of life. The scaling up of health and nutrition interventions in the first 1000 days is associated with substantial reductions in child malnutrition and mortality.^2-4^ These include interventions from preconception to early childhood.^3-6^ Multiple studies have tracked the uptake of these interventions in low- and middle-income countries (LMICs).^3^^,^^7-12^

A measure that is widely used to track uptake is coverage (proportion of individuals who received an intervention out of all eligible for it).^3^ There is guidance available on coverage estimation among eligible populations.^4^^,^^13^^,^^14^ To ensure population representative estimates, the most common method for estimating coverage is to rely on surveys that collect household and individual data on utilization of interventions. The Demographic Health Surveys (DHS) and Multiple Indicator Cluster Surveys (MICS) are typically used to assess coverage of maternal and child interventions in LMICs.^4^^,^^15^

While coverage estimates are useful for monitoring an intervention in a population, policymakers cannot directly apply these to evaluate the scale of financial, human, and infrastructural resource gaps in the delivery of an intervention. To assess these gaps, an estimate of the total population eligible for and covered by an intervention is required. As surveys interview a representative subsample of the eligible population, they cannot be used alone to estimate the total population covered by an intervention. Moreover, coverage expansion does not necessarily reflect increased utilization of an intervention. For instance, it is possible that over time, the number of children (denominator for calculating coverage) decreased due to declining fertility rates. Therefore, an increase in coverage (eg, vaccinations for children) could primarily be due to changes in the denominator rather than an increase in the number of children receiving the intervention (numerator).

In high-income countries, health systems routinely collect population data and are linked to health facilities, thus providing credible eligible population estimates for a service. However, in LMICs, due to inadequate data collection infrastructure, insufficient staff training, and inconsistent standards, health system data suffer from quality issues. Furthermore, health system data might not capture all intended beneficiaries of an intervention as data collection is often limited to public health facilities.^16^ An alternate approach is to triangulate coverage estimates with sources of demographic data such as censuses and vital statistics systems. Such an approach has proven to be difficult in LMICs, where censuses might be infrequently conducted, and vital statistics have been shown to underreport birth and mortality rates.^17^

To address these concerns, we proposed a methodology to estimate the population eligible for and covered by maternal and child health and nutrition interventions across the continuum of care. We illustrated our method using India’s National Nutrition Mission, which delivers a comprehensive and globally recommended set of health and nutrition interventions under 1 overarching program.^18^ Despite our focus on India, this methodology can be extended to other countries, as we utilized data sources that are available for most LMICs, and the interventions analyzed are delivered in most countries.^5^^,^^19^ To demonstrate external validity, we extended the methodology developed to Nepal to estimate the population eligible for and covered by maternal and child interventions at the national and subnational levels.

## Selection of interventions

**Figure 1:**
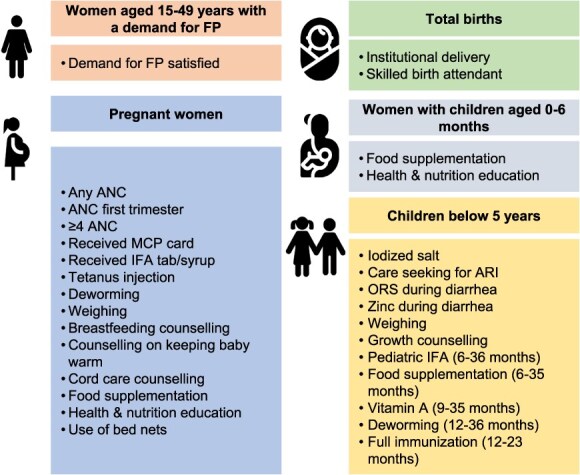
Health and nutrition interventions delivered in the first 1000 days by beneficiary group. ANC, antenatal care; ARI, acute respiratory infection; FP, family planning; IFA, iron folic acid; MCP, mother-child protection; ORS, oral rehydration salts. Adapted from Menon et al.^20^

We selected health and nutrition interventions delivered across the continuum of care using India’s National Nutrition Monitoring Framework.^20^  Figure 1 presents the selected interventions organized by the beneficiary group. The selected interventions by group include the following:

- *Women aged 15 to 49 years with a demand for contraception:* demand for family planning satisfied
- *Pregnant women:* antenatal care (ANC), receipt of a mother-child protection card (tracks services received during pregnancy, delivery, and childhood), iron folic acid supplementation, tetanus injection, deworming, weighing, counseling, food supplementation, health and nutrition education, and use of bed nets
- *Total births:* institutional delivery, skilled birth attendant
- *Women with children aged 0 to 6 months:* food supplementation, health and nutrition education
- *Children:* iodized salt, care seeking for acute respiratory infection; oral rehydration salts and zinc during diarrhea; counseling on child growth (0-59 months); pediatric iron folic acid, food supplementation (6-35 months); vitamin A (9-35 months); deworming (12-35 months); full immunization (12-23 months)

Table S1 provides definitions for the interventions.

## Data sources

### Population estimates

To illustrate our method, we used data from the United Nations’ World Population Prospect 2022 (WPP) database and fourth (2015-2016) and fifth (2019-2021) rounds of the National Family Health Survey (NFHS), India’s equivalent of the DHS.^19^^,^^21^^,^^22^ We selected 2015 and 2020 to estimate the populations eligible and covered, as these were the midpoint years of the NFHS-4 and NFHS-5, respectively.

Table 1 provides details on data sources by beneficiary group. The WPP database uses data from nationally representative surveys to estimate populations. We extracted data for India’s total population, women aged 15 to 49 years, and crude birth, maternal mortality, infant mortality, and under-5 mortality rate from the database.

**Table 1:** Data sources used for estimating population groups.

**Population group**	**Data used from World Population Prospect 2022** ^**a**^	**Data used to make adjustments from NFHS-4** ^**b**^ **and NFHS-5**^**c**^
Women aged 15-49 years with a demand for contraception	Women aged 15-49 years	Percentage of women aged 15-49 years who are in menopause or infecund, never had sex, or unmarried and not had sex in the past 30 days^d^ Percentage of currently married women aged 15-49 years who use female sterilization as a form of contraception^d^
Pregnant women	Total population Crude birth rate (per 1000)	Percentage of children aged 0-12 months who are the second or third born in a multiple birth^e^ Percentage of pregnancies that ended in stillbirth^e^ Percentage of pregnancies that ended in fetal loss^f^
Total births	Total population Crude birth rate (per 1000)	Percentage of pregnancies that ended in stillbirth^g^
Women with children aged 0-6 months	Total population Crude birth rate (per 1000) Infant mortality rate (per 1000) Maternal mortality rate (per 100,00 live births)	Percentage of children aged 0-12 months who are the second or third born in a multiple birth^e^
Children aged 0-59 months, 9-35 months, 6-35 months, and 12-35 months	Total population Crude birth rate (per 1000) Under-5 mortality (per 1000) Infant mortality rate (per 1000)	Mean mortality rate (per 1000) for each birth cohort born in the past 5 years^h^ Cumulative mortality rate (per 1000) for birth cohorts born in the past 5 years Proportion of 6-11 months deaths in total infant deaths Proportion of 9-11 months deaths in total infant deaths

a United Nations Department of Economic and Social Affairs, Population Division (2022). World Population Prospects 2022.

b IIPS (International Institute for Population Sciences). National Family Health Survey (NFHS-4), 2015-16: India. Mumbai, India; 2016.

c IIPS (International Institute for Population Sciences). National Family Health Survey (NFHS-5), 2019-21: India. Mumbai, India; 2021.

d These estimated percentages were employed to exclude women without a demand for family planning from the population estimate for women aged 15 to 49 years with a demand for family planning.

e The total live births were adjusted downward for multiple births to arrive at the estimate of women with a live birth.

f These estimated percentages were used to adjust the number women with a birth upward to obtain the number of pregnancies.

g The total live births were adjusted upward for stillbirths to obtain the total number of births.

h The mean mortality rate from the NFHS is extrapolated to the WPP to estimate the number of deaths in each birth cohort.

We estimated the proportion of women aged 15 to 49 years who would not have a demand for family planning, the proportion of women aged 15 to 49 years who use female sterilization as a form of contraception, and the proportion of pregnancies that ended in fetal loss (abortion or miscarriage) and stillbirth using the NFHS-4 (*n* = 699 686) and NFHS-5 (*n* = 724 115) women data sets. Additionally, we estimated the proportion of children who are second or third born in a multiple birth using the NFHS-4 (*n* = 259 627) and -5 (*n* = 232 920) child data sets. Lastly, we estimated the mean mortality rate for each cohort born in the past 5 years using the NFHS-4 (*n* = 250 729) and NFHS-5 (*n* = 224 433) birth record data. For proportions and prevalences estimated from NFHS data, we additionally derived the 95% confidence intervals for these estimates. We used these intervals to estimate the lower and upper bounds of the prediction intervals of population estimates when making adjustments.^23^

As the NFHS only captures diarrhea cases over a 2-week recall period, we cannot use the diarrhea prevalence from the NFHS to estimate the annual diarrhea burden. Instead, we obtained the annual diarrhea incidence for 2015 and 2020 from the Global Burden of Disease 2021.^24^

### Coverage of health and nutrition interventions

We used child-level data from the NFHS-4 (*n* = 178 874) and NFHS-5 (*n* = 170 697) to estimate the population-weighted coverage of health and nutrition interventions. As most questions about the receipt of health and nutrition interventions are asked with regard to the most recent birth in the NFHS-4 and NFHS-5, we only included the youngest child born in the 5 years preceding the survey in our sample for estimating coverage based on DHS and India’s programmatic guidelines.^20^

## Estimating beneficiary populations

We developed methodologies to estimate the eligible population for the groups of interest.

### Women aged 15 to 49 years with a demand for contraception

Equation 1 was employed to calculate the number of women with a demand for contraception:


(1) \begin{align*}&\notag Women\ with\ a\ demand\ for\ contraception= Women\ of\ repoductive\\ &\quad age- women\ in\ menopause- women\ who\ are\ infecund- women\notag\\ &\quad not\ engaged\ in\ sexual\ activity- women\ using\ female\ sterilization\dots \end{align*}

Women with a demand for family planning is defined as the number of women aged 15 to 49 years who have a met or unmet need for contraception for limiting or spacing. Therefore, women who are in menopause, are infecund, have never had sex, are unmarried, and have not had sex in the past 30 days were excluded from the population eligible for this intervention.^13^ Additionally, as sterilized women are less likely to seek out family planning services, which consist of access to various modern contraception methods in LMICs, we exclude women using female sterilization as a form of contraception from the target population.^25^ Thus, downward adjustments were made to the number of women aged 15 to 49 years to arrive at the number of women with a demand for contraception. The number of women with a demand for contraception was 161.2 million (160.0-163.7 million) in 2015 and 176.9 million (175.1-178.5 million) in 2020 (Table 2).

**Table 2:** Method for estimating number of women aged 15 to 49 years with a demand for contraception.

	**2015** ^**a**^	**2020**
Step 1: Adjusting number of WRA downward for women in menopause or infecund		
Number of women aged 15-49 years (1)	341 191 494	364 459 975
Percentage of women aged 15-49 years who are in menopause or infecund (2)	14.1 (13.9, 14.3)	9.2 (9.1, 9.3)
Number of women aged 15-49 years who are in menopause or infecund (3) Obtained by multiplying (1) and (2)	48 108 001 (47 425 618, 48 790 384)	33 530 318 (33 165 858, 33 894 778)
Number of women aged 15-49 years excluding those who are in menopause or infecund (4) Obtained by subtracting (3) from (1)	293 083 493 (292, 401 110, 293 765 876)	330 929 657 (330 565 197, 331 294 117)
Step 2: Adjusting number of WRA for women who have never had sex or are unmarried and not had sex in the past 30 days		
Percentage of women who have never had sex or are unmarried and not had sex in the past 30 days (5)	23.7 (23.5, 23.9)	24.7 (24.6, 24.9)
Number of women who have never had sex or are unmarried and not had sex in the past 30 days (6) Obtained by multiplying (4) and (5)	69 460 788 (68 714 261, 70 210 044)	81 739 826 (81 319 038, 82 492 235)
Number of women aged 15-49 years who have a demand for contraception (7) Obtained by subtracting (6) from (4)	223 622 705 (222 191 066, 225 051 615)	249 189 831 (248 072 962, 249 975 079
Step 3: Adjusting women aged 15-49 years who have an active demand for contraception downward for women using female sterilization as a form of contraception		
Percentage of women (95% CI) aged 15-49 years who are using female sterilization as form of contraception (8)	27.9 (27.6, 28.1)	29.0 (28.8, 29.2)
Number of women aged 15-49 years who are using female sterilization as a form of contraception (9) Obtained by multiplying (7) with (8)	62 390 735 (61 324 734, 63 239 504)	72 265 051 (71 445 013, 72 992 723)
Number of women aged 15-49 years with a demand for contraception Obtained by subtracting (9) from (7)	161 231 970 (158 951 562, 163 726 881)	176 924 780 (175 080 239, 178 530 066)

Estimates for women aged 15 to 49 years for 2015 and 2020 were obtained from the UN World Population Prospect database. Estimates for percentage of women in menopause or infecund, percentage of women who have never had sex or unmarried and not had sex in the past 30 days, and number of women who are currently using female sterilization as a form of contraception obtained from NFHS-4 (2015-2016) and NFHS-5 (2019-2021) women data. The years 2015 and 2020 were selected for the population estimation as these were the midpoints for the NFHS-4 and NFHS-5 survey, respectively.

a The 95% confidence intervals reported for percentage estimates from NFHS-4 and NFHS-5. Prediction intervals for population estimates obtained by multiplying WPP population estimates (1) with lower and upper bounds of the 95% confidence interval (2). Thereafter, prediction intervals were obtained by multiplying lower and upper bounds of the 95% confidence interval of the percentage estimates with lower and upper bounds of prediction intervals of the population estimates, respectively. WRA: Women of reproductive age

### Pregnant women

The number of women who had a pregnancy that resulted in a birth is the preferred estimate of the population eligible for pregnancy interventions, as these women are most likely to seek our health services across all 3 trimesters.^26^

Equation 2 was employed to calculate the number of women who had a pregnancy that resulted in a birth:


(2) \begin{align*}& Number\ of\ women\ who\ had\ a\ pregnancy\ that\ resulted\ in\notag\\&\quad birth= Number\ of\ live\ births- number\ of\ multiple\ births+ number\notag\\&\quad of\ stillbirths\dots \end{align*}

First, the number of live births was calculated by multiplying the crude birth rate by the total population. Second, the number of live births was adjusted downward to account for the proportion of multiple births to arrive at the number of women who had a live birth. Third, the number of women who had a live birth was adjusted upward for the percentage of pregnancies that ended in a stillbirth using the following equation:


$$ {B}_l=\left(1-s\right)\ast{B}_t $$

(3) \begin{equation*} {B}_t=\frac{B_l}{\left(1-s\right)}\dots \end{equation*}

Here, ${B}_l$ is the number of women who had a birth that resulted in a live birth, $\left(1-s\right)$ is the proportion of live births in the total number of births, and ${B}_t$ is the total number of women who had a birth. $s$ is the proportion of woman who had a birth that resulted in a stillbirth.

The number of women with a pregnancy that resulted in a birth was 24.9 million (24.9-24.9 million) in 2015 and 23.3 million (23.3-23.3 million) in 2020 (Table 3).

**Table 3:** Method for estimating number of pregnant women.

	**2015** ^**a**^	**2020**
Step 1: Estimating number of live births		
Crude birth rate (per 1000) (1)	18.8	16.6
Total population (2)	1 322 866 505	1 396 387 127
Number of live births (3) Obtained by multiplying (1) and (2) and dividing by 1000	24 823 590	23 140 927
Step 2: Adjusting number of live births downward for second and third born in multiple births to obtain number of women who had a live birth		
Percentage of children aged 0-12 months who are second or third born in multiple births (4)	0.8 (0.7, 0.9)	0.8 (0.7, 0.9)
Number of live births who are second or third born in multiple births (5) Obtained by multiplying (3) and (4)	198 589 (173 765, 223 412)	175 871 (161 986, 208 268)
Number of women who had a live birth (6) Obtained by subtracting (5) from (3)	24 625 001 (24 600 178, 24 649 825)	22 965 056 (22 932 659, 22 978 941)
Step 3: Adjusting number of women who had a live birth upward for stillbirths to obtain number of women who had a pregnancy that resulted in a birth		
Percentage of pregnancies that ended in stillbirth (7)	1.4 (1.3, 1.5)	1.3 (1.3, 1.4)
Adjusting (6) upward for number of pregnancies that ended in stillbirths to obtain number of women who had a pregnancy that resulted in birth (8) Obtained by dividing (6) by (100%-(7))	24 974 646 (24 949 342, 24 974 800)	23 267 534 (23 257 666, 23 281 602)

The 2015 and 2020 estimates for crude birth rate and total population were obtained from the UN World Population Prospects database. Estimates for percentage of children 0 to 12 months who are second or third born in multiple births, percentage of pregnancies that ended in stillbirth, and percentage of pregnancies that ended in abortion or miscarriage obtained from the NFHS-4 (2015-2016) and NFHS-5 (2019-2021) women data. The years 2015 and 2020 were selected for the population estimation as these were the midpoints for the NFHS-4 and NFHS-5 survey, respectively.

a The 95% confidence intervals reported for percentage estimates from NFHS-4 and NFHS-5. Prediction intervals for population estimate of number of live births that are second or third born in multiple births obtained by multiplying lower and upper bounds of the interval of the percentage estimate of children who are second or third born in multiple births (4) with the population estimate for the number of live births (3). For the prediction interval of the number of women who had a live birth (4), the upper bound of (5) is subtracted from (3) to obtain the lower bound, and the lower bound of (5) is subtracted from (3) to obtain the upper bound. For the prediction interval of (8), the lower and upper bounds of the 95% confidence interval were subtracted from 100%. The lower and upper bounds of (6) were then divided by the resulting percentage to obtain the lower and upper bounds of (8).

### Total births

The number of total births was estimated by adjusting the number of live births upward for stillbirth, assuming that pregnancies that ended in a stillbirth are eligible for interventions during delivery. Equation 4 was employed to calculate the number of total births:


(4) \begin{equation*} Total\ births= Number\ of\ live\ births+ number\ of\ stillbirths\dots \end{equation*}

The number of total births was 25.2 million (25.2-25.2 million) in 2015 and 23.4 million (23.4-23.5 million) in 2020 (Table 4).

**Table 4:** Method for estimating number of total births.

	**2015** ^**a**^	**2020**
Step 1: Estimating number of live births		
Crude birth rate (per 1000) (1)	18.8	16.6
Total population (2)	1 322 866 505	1 396 387 127
Number of live births (3) Obtained by multiplying (1) and (2) and dividing by 1000	24 823 590	23 140 927
Step 2: Adjusting number of live births upward for number of stillbirths to obtain number of total births		
Percentage of pregnancies that ended in still births (4)	1.4 (1.3, 1.5)	1.3 (1.3, 1.4)
Number of total births Obtained by dividing (3) by (100%-(4))	25 176 055 (25 150 547, 25 201 614)	23 445 721 (23 445 721, 23 469 500)

The 2015 and 2020 estimates for crude birth rate and total population were obtained from the UN World Population Prospects database. The years 2015 and 2020 were selected for the population estimation as these were the midpoints for the NFHS-4 and NFHS-5 survey, respectively. Percentage of pregnancies that ended in stillbirth was estimated using NFHS-4 and NFHS-5 women data.

a The 95% confidence intervals reported for percentage estimates from NFHS-4 and NFHS-5. Prediction intervals for estimate of total births obtained by subtracting the lower and upper bounds of the 95% confidence interval of (4) from 100%. The resulting percentage was then divided by the number of live births (3).

### Women with children aged 0 to 6 months

Equation 5 was used to estimate the number of women with children aged 0 to 6 months:


(5) \begin{align*}& Women\ with\ children\ aged\notag\\&\quad 0-6\ months=\frac{\begin{array}{c}Live\ births- live\ births\ resulting\ in\ maternal\\ death- number\ of\ multiple\ live\ births\end{array}}{2}\dots \end{align*}

First, the number of live births was adjusted downward for maternal mortality to arrive at the number of live births that did not result in maternal death. Second, the number of live births that did not result in maternal death was further adjusted downward for the proportion of multiple live births to estimate the number of women who had a live birth that did not result in their death. Lastly, assuming that half of the women who survived childbirth in the year gave birth in the past 6 months, the number of women who survived childbirth was divided by 2. The number of women with children aged 0 to 6 months arrived at was 12.3 million (12.3-12.3 million) for 2015 and 11.5 million (11.5-11.5 million) for 2020 (Table 5).

**Table 5:** Method for estimating number of women with children aged 0 to 6 months.

	2015^**a**^	2020
Step 1: Estimating number of live births		
Crude birth rate (per 1000) (1)	18.8	16.6
Total population (2)	1 322 866 505	1 396 387 127
Number of live births (3) Obtained by multiplying (1) and (2) and dividing by 1000	24 823 590	23 140 927
Step 2: Adjusting number of live births downward for maternal mortality to obtain number of live births that did not result in maternal death		
Maternal mortality ratio (per 100 000 live births) (4)	121.9	92.7
Number of live births that resulted in maternal death (5) Obtained by multiplying (3) by (4) and dividing the product by 100 000	30 260	21 452
Number of live births that did not result in maternal death (6) Obtained by subtracting (5) from (3)	24 793 330	23 119 475
Step 3: Adjusting number of live births downward for second and third born in multiple births to obtain number of women who had a live birth and did not die in childbirth		
Percentage of living children aged 0-12 months who are second or third born in multiple births (7)	0.8 (0.7, 0.9)	0.8 (0.7, 0.9)
Number of live births who are second or third born in multiple births (8) Obtained by multiplying (6) and (7)	198 347 (173 553, 223 140)	184 956 (161 836, 208 075)
Number of women who had a live birth who did not die in childbirth (9) Obtained by subtracting (8) from (6)	24 594 983 (24 570 190, 24 619 777)	22 934 519 (22 911 400, 22 957 639)
Step 4: Number of living women who had a live birth in the past 6 months		
Number of living women who had a live birth in the past 6 months Obtained by dividing (9) by 2	12 297 492 (12 285 095, 12 309 889)	11 467 260 (11 455 700, 11 478 820)

The 2015 and 2020 estimates for crude birth rate, total population, and maternal mortality rate were obtained from the UN World Population Prospect database. Estimates of the percentage of 0 to 12 months who are second or third born in multiple births were obtained from NFHS-4 (2015-2016) and NFHS-5 (2019-2021) women data. The years 2015 and 2020 were selected for the population estimation as these were the midpoints for the NFHS-4 and NFHS-5 survey, respectively.

a The 95% confidence intervals reported for percentage estimates from NFHS-4 and NFHS-5. The prediction interval for the number of live births that are second or third born in multiple births (8) was obtained by multiplying the lower and upper bounds of (7) by (6). The prediction interval for the number of women who did not die in childbirth (9) was obtained by subtracting the upper bound from (6) for the lower bound and subtracting the lower bound from (6) for the upper bound. The prediction interval for the number of living women who had a live birth in the past 6 months was obtained by dividing the lower and upper bounds of the prediction interval of (9) by 2.

### Children

Health and nutrition interventions relevant for children target different age groups, depending on the programmatic guidelines for the intervention. As a result, we required population estimates for the following age groups for India: 0 to 59, 0 to 12, 6 to 35, 9 to 35, and 12 to 35 months. For full immunization, the relevant age group for coverage estimation is children aged 12 to 23 months to capture the percentage of children who have been exposed to an age-appropriate vaccination schedule. However, we selected children aged 0 to 11 months as the intended beneficiary population as these children will be the ones targeted by vaccinations included in the national vaccination schedule.

The WPP database provides data on infant and under-5 mortality rates but does not provide the birth cohort–specific cumulative mortality rates for children older than 11 months. Cohort-specific death estimates were needed to estimate the number of living children in each birth cohort. However, children die at different rates across age groups in early childhood, with the highest death rates in infancy and lower death rates thereafter.^27^

Cohort cumulative mortality rates were estimated for children older than 11 months by extrapolating the patterns observed in the mortality rates from the NFHS to the infant mortality rate in the WPP (Table 6) using the following proportional equivalence.


\begin{align*}& Cumulative\ cohort\ specific\ mortality\ rates\ in\ NFHS\notag\\&\quad\approx Cumulative\ cohort\ specific\ mortality\ rates\ in\ WPP \end{align*}

**Table 6:** Cohort-specific mortality rates, number of live births, and number of living children in each birth cohort.

Birth cohort	Cohort mortality rate (per 1000)^a^	Proportion of cumulative mortality rate (1)	Estimated cohort mortality rate (2)	Estimated cumulative mortality rate by cohort (3)	Number of live births^b^ (4)	Number of deaths (5)	Number of living children
	Source: NFHS-4 and NFHS-5	Obtained by dividing cohort mortality rate by cumulative mortality rate	Obtained by multiplying (1) by WPP’s estimate of U5MR	Obtained by cumulatively adding mortality rates in (2) by cohort	Source: Author’s calculation using WPP data	Obtained by multiplying (3) by (4)	Obtained by subtracting (5) from (4)
2015							
0-11	0.0414 (0.0391, 0.0437)	0.83 (0.80, 0.86)	0.0350	0.0350	24 823 590	868 826	23 954 764
12-23	0.0031 (0.0025, 0.0037)	0.06 (0.05, 0.07)	0.0027 (0.0018, 0.0024)	0.0377 (0.0368, 0.0374)	24 901 739	938 106 (916 384, 931 325)	23 963 633 (23 032 308, 23 985 355)
24-35	0.0020 (0.0014, 0.0025)	0.04 (0.03, 0.05)	0.0017 (0.0012, 0.0018)	0.0393 (0.0380, 0.0392)	25 738 718	1 012 697 (978 071, 1 008 958)	24 726 021 (24 729 760, 24 760 647)
36-47	0.0021 (0.0016, 0.0027)	0.04 (0.04, 0.05)	0.0018 (0.0014, 0.0018)	0.0412 (0.0394, 0.0413)	26 026 303	1 071 885 (1 025 436, 1 074 886)	24 954 418 (24 951 417, 25 000 867)
48-59	0.0015 (0.0010, 0.0020)	0.03 (0.02, 0.04)	0.0013 (0.0007, 0.0014)	0.0425 (0.0401, 0.0426)	26 340 876	1 119 301 (1 056 269, 1 122 121)	25 221 575 (25 218 755, 25 284 607)
Cumulative/U5MR	0.0502 (0.0456, 0.0546)		0.0430				
2020							
0-11	0.0334 (0.0312, 0.0356)	0.85 (0.82, 0.90)	0.0283	0.0283	23 140 927	654 888	22 486 039
12-23	0.0023 (0.0017, 0.0029)	0.06 (0.05, 0.07)	0.0019 (0.0015, 0.0020)	0.0302 (0.0298, 0.0303)	23 580 677	712 982 (702 704. 714 495)	22 867 695 (22 153 200, 22 877 973)
24-35	0.0008 (0.0005, 0.0011)	0.02 (0.01, 0.03)	0.0007 (0.0003, 0.0008)	0.0309 (0.0301, 0.0311)	24 164 277	746 787 (727 345, 751 509)	23 417 490 (23 412 768, 23 436 932)
36-47	0.0017 (0.0012, 0.0022)	0.04 (0.03, 0.05)	0.0014 (0.0008, 0.0014)	0.0323 (0.0309, 0.0325)	24 254 999	783 794 (783 436, 788 287)	23 471 205 (23 466 712, 23 471 563)
48-59	0.0011 (0.0007, 0.0012)	0.03 (0.02, 0.03)	0.0010 (0.0006, 0.0010)	0.0333 (0.0315, 0.0335)	24 783 513	825 291 (780 681, 830 248)	23 958 222 (23 953 265, 24 002 832)
Cumulative/U5MR	0.0393 (0.0353,0.0430)		0.03300				

Abbreviations: NFHS, National Family Health Survey; WPP, World Population Prospects; U5MR, Under five mortality rates.

The 2015 and 2020 estimates for infant mortality rate, under-5 mortality rate, crude birth rate, and total population were obtained from the UN’s World Population Prospect database. The 2015 and 2020 estimates for cohort mortality rates were obtained from NFHS-4 (2015-2016) and NFHS-5 (2019-2021) birth record data. The years 2015 and 2020 were selected for the population estimation as these were the midpoints for the NFHS-4 and NFHS-5 survey, respectively.

a The 95% confidence intervals reported for percentage estimates from NFHS-4 and NFHS-5. The prediction interval for the proportion of cumulative mortality (1) was obtained by dividing the lower and upper bounds of the 95% confidence interval for the cohort mortality rate by the lower and upper bounds of the 95% confidence interval of the cumulative/U5MR, respectively. The lower and upper bounds of the prediction interval for the estimated cohort mortality rate (2) were obtained by multiplying the lower and upper bounds of (1) with the U5MR from the WPP, respectively. The prediction interval for the estimated cumulative mortality rate by cohort (3) was obtained by cumulatively adding the prediction intervals of the mortality rates by cohort. The prediction interval for the number of deaths (5) was obtained by multiplying the prediction interval of (3) by the number of live births (4). The prediction interval of the number of living children was obtained by subtracting the upper bound of (5) from (4) for the lower bound and subtracting the lower bound of (5) from (4) for the upper bound.

b The number of live births for each birth cohort was obtained by multiplying the total population by the crude birth rate from the WPP. Table S2 estimates the number of live births from 2011 to 2020.

The NFHS birth record data provide information on the imputed age in months at death for children. Using this information, the mean mortality rate per 1000 live births in the year preceding the survey was estimated for each cohort born in the past 5 years using the package *syncmrates* that estimates the cohort-specific mean mortality rates by using a synthetic cohort life table approach.^28^ Additionally, the cumulative mortality rate for children younger than 5 years was estimated by summing the cohort-specific mortality rates. For each cohort, the proportion of the cohort’s mortality rate in the cumulative mortality rate was estimated. The infant mortality rate from the WPP is applied to the cohort aged 0 to 11 months as this cohort would have been fully exposed to the infant mortality. For the remaining cohorts, the proportions calculated for each birth cohort were applied to the under-5 mortality rate from the WPP based on the assumption of proportional equivalence. The cohort mortality rates are then summed by each cohort to derive the cohort cumulative mortality rate.

For each cohort, the number of deaths was obtained using Equation 6:


(6) \begin{align*} \notag &Total\ deaths= Number\ of\ live\ births\times Cohort\ specific\ cumulative\\ &\quad mortality\ rate\dots \end{align*}

The number of live births for each cohort was estimated by multiplying the total population in each year by the crude birth rate (Table S2). The cohort cumulative mortality rate was applied to the number of live births to estimate the number of deaths in each cohort.

For each cohort, the number of living children is obtained using Equation 7 (Table 6):


(7) \begin{equation*} Total\ living\ children= Number\ of\ live\ births- Total\ deaths\dots \end{equation*}

To estimate the number of children aged 6 to 11 and 9 to 11 months who were needed to calculate the number of children ages 6 to 35 and 9 to 35 months, respectively, we estimated the proportion of deaths among infants aged 6 to 11 months and 9 to 11 months in the total infant deaths. We then applied these proportions to the total infant deaths to calculate the number of deaths in infants aged 6 to 11 and 9 to 11 months, respectively. Next, we subtract these from the number of infants aged 6 to 11 months (assumed to be half of the live births in the cohort aged 0-11 months) and 9 to 11 months (assumed to be three-fourths of the live births in the cohort aged 0-11 months) to arrive at the number of children aged 6 to 11 and 9 to 11 months, respectively (Table S3).

Using the number of living children in each cohort born in the past 5 years, the number of living children aged 0 to 59, 6 to 35, 9 to 35, and 12 to 35 months was estimated (Table S4).

### Estimating eligible populations covered by interventions

For each intervention, the number of beneficiaries covered is calculated using Equation 8:


(8) \begin{align*}\notag & Total\ beneficiaries\ covered= Coverage\ proportion\times Total\ eligible\\&\quad population\dots \end{align*}

Additionally, the lower and upper bounds of the 95% confidence interval of the weighted coverage estimate were multiplied by the beneficiary population estimate to arrive at the 95% prediction interval for the mean population covered.

## Illustration 1: national estimates of eligible populations in India

Table 7 reports the mean population covered by intervention, along with the estimated change in the mean population covered between 2015 and 2020. For example, the number of pregnant women eligible for health and nutrition services was 24.9 (24.9-24.9) and 23.3 (23.3-23.3) million in 2015 and 2020, respectively. The coverage for ≥4 ANC visits was 50.7% (50.2-51.1%) in 2015 and 55.2% (54.8-55.6%) in 2020. Multiplying the population estimate by the coverage estimate, we find that the number of eligible pregnant women who received ≥4 ANC visits was 12.6 (12.5-12.7) million in 2015 and 12.9 (12.8-13.0) million in 2020. In this example, the decline in the number of pregnant women (1.7 million) was larger than the increase in the number of women receiving ≥4 ANC (0.2 [0.0-0.5] million), suggesting that the observed increase in coverage was partially driven by a decline in the eligible population.

**Table 7:** Beneficiary population covered by each health and nutrition intervention across the continuum of care in India.

		2015			2020			Estimated change in population covered (mn)
		Coverage estimate,^**a**^ %	Population estimate (mn)	Population covered estimate (mn)	Coverage estimate, %	Population estimate (mn)	Population covered estimate (mn)	
Beneficiary group	Intervention	Mean (95% CI)	Mean (PI)	Mean (PI)	Mean (95% CI)	Mean (PI)	Mean (PI)	Mean (PI)
Women aged 15-49 years with a demand for FP	Demand for FP satisfied by modern methods	51.8 (51.4, 52.3)	161.2 (159.0, 163.7)	83.5 (81.7, 85.6)	59.1 (58.7, 59.6)	176.9 (175.8, 178.5)	104.5 (103.2, 106.4)	21.0 (17.6, 24.7)
Pregnant women	Any ANC	80.2 (79.8, 80.5)	24.9 (24.9, 24.9)	20.0 (19.9, 20.0)	85.3 (85.0, 85.6)	23.3 (23.3, 23.3)	19.9 (19.8, 19.9)	–0.1 (–0.2, 0.1)
	ANC first trimester	56.7 (56.3, 57.2)	24.9 (24.9, 24.9)	14.1 (14.0, 14.2)	64.1 (63.7, 64.5)	23.3 (23.3, 23.3)	14.9 (14.8, 15.0)	0.8 (0.6, 1.0)
	≥4 ANC	50.7 (50.2, 51.1)	24.9 (24.9, 24.9)	12.6 (12.5, 12.7)	55.2 (54.8, 55.6)	23.3 (23.3, 23.3)	12.9 (12.8, 13.0)	0.2 (0.0, 0.5)
	Received MCP card	89.6 (89.3, 89.9)	24.9 (24.9, 24.9)	22.3 (22.2, 22.4)	96.0 (95.8, 96.2)	23.3 (23.3, 23.3)	22.4 (22.3, 22.4)	0.1 (–0.1, 0.2)
	Received IFA tab/syrup	78.5 (78.2, 78.8)	24.9 (24.9, 24.9)	19.5 (19.5, 19.6)	87.8 (87.5, 88.1)	23.3 (23.3, 23.3)	20.5 (20.4, 20.5)	0.9 (0.8, 1.1)
	Neonatal tetanus	89.1 (88.9, 89.4)	24.9 (24.9, 24.9)	22.2 (22.1, 22.3)	91.5 (91.3, 91.7)	23.3 (23.3, 23.3)	21.3 (21.3, 21.4)	–0.9 (–1.0, –0.8)
	Deworming	18.3 (17.9, 18.7)	24.9 (24.9, 24.9)	4.6 (4.5, 4.7)	31.2 (30.8, 31.6)	23.3 (23.3, 23.3)	7.3 (7.2, 7.4)	2.7 (2.5, 2.9)
	Weighing	76.7 (76.3, 77.0)	24.9 (24.9, 24.9)	19.1 (19.0, 19.2)	91.3 (91.0, 91.5)	23.3 (23.3, 23.3)	21.3 (21.2, 21.3)	2.2 (2.0, 2.3)
	Breastfeeding counseling	40.7 (40.3, 41.2)	24.9 (24.9, 24.9)	10.1 (10.0, 10.3)	62.4 (61.9, 62.8)	23.3 (23.3, 23.3)	14.5 (14.4, 14.6)	4.4 (4.2, 4.6)
	Counseling on keeping baby warm	38.4 (37.9, 38.8)	24.9 (24.9, 24.9)	9.6 (9.4, 9.7)	59.9 (59.4, 60.4)	23.3 (23.3, 23.3)	14.0 (13.8, 14.1)	4.4 (4.2, 4.6)
	Cord care counseling	36.3 (35.8, 36.7)	24.9 (24.9, 24.9)	9.0 (8.9, 9.1)	58.3 (57.9, 58.8)	23.3 (23.3, 23.3)	13.6 (13.5, 13.7)	4.5 (4.4, 4.6)
	Food supplementation	53.1 (52.7, 53.6)	24.9 (24.9, 24.9)	13.2 (13.1, 13.3)	67.2 (66.7, 67.6)	23.3 (23.3, 23.3)	15.7 (15.5, 15.8)	2.4 (2.2, 2.6)
	Health and nutrition education	39.9 (39.4, 40.3)	24.9 (24.9, 24.9)	9.9 (9.8, 10.0)	60.0 (59.5, 60.2)	23.3 (23.3, 23.3)	14.0 (13.9, 14.0)	4.0 (3.8, 4.2)
	Use of bed nets	53.9 (53.5, 54.3)	24.9 (24.9, 24.9)	13.4 (13.3, 13.5)	55.7 (55.2, 56.1)	23.3 (23.3, 23.3)	13.0 (12.9, 13.1)	–0.4 (–0.7, –0.3)
Total births	Institutional delivery	81.9 (81.5, 82.2)	25.2 (25.2, 25.2)	20.6 (20.5, 20.7)	90.2 (90.0, 90.5)	23,4 (23.4, 23.5)	21.1 (21.1, 21.3)	0.5 (0.3, 0.7)
	Skilled birth attendant	84.1 (83.8, 84.4)	25.2 (25.2, 25.2)	21.2 (21.1, 21.3)	90.8 (90.5, 91.0)	23,4 (23.4, 23.5)	21.2 (21.2, 21.4)	0.1 (–0.1, 0.3)
Women with children aged 0-6 months	Food supplementation	48.9 (48.4, 49.4)	12.3 (12.3, 12.3)	6.0 (6.0, 6.1)	64.0 (63.5, 64.5)	11.5 (11.5, 11.5)	7.4 (7.3, 7.4)	1.4 (1.3, 1.4)
	Health and nutrition education	36.0 (35.6, 36.4)	12.3 (12.3, 12.3)	4.4 (4.4, 4.5)	56.4 (55.9, 56.9)	11.5 (11.5, 11.5)	6.5 (6.4, 6.5)	2.1 (2.0, 2.1)
Children aged 0-11 months	Full immunization	63.0 (62.4, 63.7)	24.0 (24.0, 24.0)	15.1 (15.0, 15.3)	76.5 (75.9, 77.1)	22.5 (22.5, 22.5)	17.2 (17.1, 17.3)	2.1 (1.8, 2.4)
Children aged 6-35 months	Pediatric IFA	26.4 (25.9, 26.9)	60.6 (59.6, 60.7)	16.0 (15.4, 16.3)	38.0 (37.5, 38.5)	57.5 (56.7, 57.5)	21.9 (21.3, 22.1)	5.9 (4.9, 6.7)
	Food supplementation	56.0 (55.5, 56.6)	60.6 (59.6, 60.7)	33.9 (33.1, 34.4)	70.8 (70.2, 71.3)	57.5 (56.7, 57.5)	40.7 (39.8, 41.0)	6.8 (5.4, 7.9)
Children aged 9-35 months	Vitamin A	62.8 (62.3, 63.4)	66.6 (65.7, 66.7)	41.8 (40.9, 42.3)	67.7 (67.1, 68.2)	63.1 (62.4, 63.1)	42.7 (41.9, 43.0)	0.9 (–0.4, 2.1)
Children aged 12-35 months	Deworming	33.1 (32.5, 33.7)	48.7 (47.8, 48.7)	16.1 (15.5, 16.4)	42.4 (41.9, 43.0)	46.3 (45.6, 46.3)	19.6 (19.1, 19.9)	3.5 (2.7, 4.4)
Children with diarrhea	ORS during diarrhea	50.7 (49.7, 51.7)	131.0 (106.1, 158.8)	66.4 (52.7, 82.1)	60.4 (59.3, 61.6)	88.3 (72.2, 105.6)	53.3 (42.8, 65.0)	–13.1 (–39.3, 12.3)
	Zinc during diarrhea	20.2 (19.4, 21.0)	131.0 (106.1, 158.8)	26.5 (20.6, 33.3)	30.6 (29.5, 31.7)	88.3 (72.2, 105.6)	27.0 (21.3, 33.5)	0.5 (–12.0, 12.9)
Children aged <5 years	Weighing	45.0 (44.5, 45.4)	122.8 (121.9, 123.0)	55.3 (54.2, 55.8)	60.9 (60.4, 61.4)	116.2 (115.5, 116.3)	70.8 (69.8, 71.4)	15.5 (13.9, 17.2)
	Counseling on child growth	28.8 (28.4, 29.3)	122.8 (121.9, 123.0)	35.4(34.6, 36.0)	45.6 (45.2, 46.1)	116.2 (115.5, 116.3)	53.0 (52.2, 53.6)	17.6 (16.2, 19.0)

Abbreviations: ANC, antenatal care; ARI, acute respiratory infection; FP, family planning; IFA, iron folic acid; MCP, mother-child protection; mn, millions; ORS, oral rehydration salts; PI, prediction interval.

Woman–last child unit-level NFHS-4 and NFHS-5 data used to estimate weighted coverage of health and nutrition interventions in 2015-2016 and 2019-2021, respectively.

Beneficiary population estimates are for the years 2015 and 2020 as these are midpoint years of the NFHS-4 and NFHS-5, respectively.

a The 95% confidence interval reported for coverage estimates from NFHS-4 and NFHS-5. The prediction interval was reported for the population estimate of the beneficiary populations. The prediction interval for the population covered estimate was obtained by multiplying the 95% confidence interval of the coverage estimate with the prediction interval of the population estimate. The prediction interval for the estimated change in the population covered was obtained by subtracting the upper bound for the 2015 estimate from the lower bound for the 2020 estimate for the lower bound and subtracting the lower bound for the 2015 estimate from the upper bound for the 2020 estimate for the upper bound.

As another illustration, coverage of weighing during childhood increased from 45.0% (44.5%-45.4%) in 2015 to 60.9% (60.4%-61.4%) in 2020. Over the same period, the number of children below 5 years declined from 122.8 (121.9-123.0) million in 2015 to 116.2 (115.5-116.3) million in 2020. The mean number of children below 5 years who were weighed in 2015 was 55.3 (54.2-55.8) million in 2015 and 70.8 (69.8-71.4) million in 2020. Therefore, in this case, the increase in coverage (15.4 percentage point (pp)) was able to offset the decline in the number of children (6.7 million). The observed coverage increase reflects an expansion in service utilization among the eligible population despite a decline in the population.

### Sensitivity checks

We performed sensitivity checks on the population estimates for women with a demand for contraception and pregnant women.

In the case of women with a demand for contraception, we had *not excluded* women who did not wish to have additional children from our estimate, as these women would still seek out family planning services to prevent further pregnancies. However, it is possible that certain family planning interventions may intend to only target women who wish to have additional children. Therefore, we further adjust the demand for contraception downward for women who do not wish to have additional children (Table S5). As more than half of the women with a demand for contraception do not wish to have children, the population estimate reduces substantially (161.2 to 72.4 million in 2015 and 176.9 to 81.6 million in 2020). On the other hand, it is possible that women who have been sterilized might seek out abortion services due to a failed sterilization, although these services are typically not part of family planning packages in LMICs.^25^ When we do not exclude sterilized women from the estimate, the women with a demand for contraception increases (223.6 million in 2015 and 249.2 million in 2020).

For estimating the number of pregnant women, we assumed that pregnant women who had a birth would be the intended beneficiary population, as these women would be likelier to seek out antenatal services. We do not make upward adjustments for women who experience fetal loss, as women typically miscarry prior to discovering their pregnancy, and women who choose to abort their fetus are less likely to seek out antenatal services. As a sensitivity check, we further adjusted the number of pregnant women who had a birth upward for fetal loss (Table S6). This increases the estimate by 4.1 million in 2015 and 4.2 million in 2020. However, we caution against using this number as the true estimate of total pregnancies in a given year, as women might not be aware they have experienced a miscarriage or choose not to report their abortion while being surveyed.^29^^,^^30^

## Illustration 2: subnational estimates of eligible populations in Nepal

To assess the applicability of our method to subnational regions, we applied the proposed methodology to Nepal and estimated the beneficiary populations at the national and ecological zone (mountain, hill, plain) levels. The 2021 Nepal census provides disaggregated population numbers at these levels.^31^ We assumed proportional equivalence between the 2021 census and the 2022 population projections to estimate the beneficiary populations at the ecological zone level (Tables S7-S13). For instance, we assumed that the proportion of women aged 15 to 49 years at the mountain level was constant between 2021 and 2022 and applied the proportion from the census to the national projection from the WPP to obtain the number of women aged 15 to 49 years residing in the mountain zone (Table S7). The Nepal Demographic Health Survey (2022) is representative at the ecological zone level, so we obtained national and ecological zone estimates from it for fertility and mortality indicators.^32^

A similar method can be applied to India using the proportions from the 2011 census, although there might have been considerable changes in the proportions since 2011, and therefore, any subnational projections using older censuses should be viewed with this caution.

### Extension to other LMICs

In Figure S1, we documented the availability of censuses and demographic surveys (DHS and MICS) for LMICs. Most countries have either the DHS or MICS conducted in the past 10 years. While WPP projections are available for all LMICs, researchers may prefer to use population estimates from the censuses for their own projections.

It is important to note that country-specific population and demographic surveys that are not captured in this documentation can also be used for projections.

## Limitations

The proposed methods are not without limitations. First, the methods rely on the availability of population representative surveys that collect demographic and coverage information. These surveys are expensive and time-consuming and, therefore, are not conducted at a high frequency in most LMICs. Data on populations utilizing a service might become critical to have on a regular basis during disruptions to services such as COVID-19. However, the methods proposed in this study can be used alongside health system data to inform policymakers on service utilization. For instance, health system data can be modeled on the estimates derived from population representative surveys to project trends in the eligible population covered. Second, the methods proposed in this article might not be easily amenable to extrapolation to subnational settings without reliable data. Through the example of Nepal, which had a recently conducted census, we estimated beneficiary populations at the subnational level. Another approach is to couple the national-level estimates with geospatial methods in a small sample enumeration approach to estimate populations at the subnational unit of interest. Such methods have been employed to estimate eligible populations for vaccinations in Africa.^33^

The methods applied to estimate the eligible populations in this study rely on assumptions. In the case of estimating the number of women with children aged 0 to 6 months, we assumed that exactly half the women who had a live birth in the previous year had a birth in the past 6 months, which does not account for changes in monthly birth rates. In India, the number of births typically peaks between August and October and dips between February and March.^34^ Finally, the uncertainty in some estimations, such as the maternal mortality rate, must be acknowledged with different modeling approaches, producing different estimates. However, LMICs often have limited capacity to collect data on maternal mortality, making modeled estimates the only source in certain cases.^35^

## Conclusion

This article proposes generalizable methods that can be applied to estimate the populations eligible for and covered by health and nutrition interventions from preconception to childhood across countries. Coverage and population estimates should be complementary, but coverage estimates are the primary focus due to the convenience in estimating them. Our methods allow researchers to easily extend coverage estimates (measured as a proportion) to the number of eligible beneficiaries in the population covered for a recommended set of interventions.

## Supplementary Material

Web_Material_kwae469

## Data Availability

Data sources used in this study are available in the public domain. The UN World Population Prospects database can be accessed here: https://population.un.org/wpp/Download/Standard/MostUsed/. The DHS data sets can be requested and accessed here: https://www.dhsprogram.com/.

## References

[1] World Health Organization, World Bank . Tracking Universal Health Coverage 2023 Global Monitoring Report. World Health Organization; 2023.

[2] Bhutta ZA, Das JK, Rizvi A, et al. Evidence-based interventions for improvement of maternal and child nutrition: what can be done and at what cost? *Lancet*. 2013;382(9890):452-477. 10.1016/S0140-6736(13)60996-4 23746776

[3] Boerma T, Requejo J, Victora CG, et al. Countdown to 2030: tracking progress towards universal coverage for reproductive, maternal, newborn, and child health. *Lancet*. 2018;391(10129):1538-1548. 10.1016/S0140-6736(18)30104-1 29395268

[4] Victora C, Requejo J, Boerma T, et al. Countdown to 2030 for reproductive, maternal, newborn, child, and adolescent health and nutrition. *Lancet Glob Health*. 2016;4(11):e775-e776. 10.1016/S2214-109X(16)30204-2 27650656

[5] Keats EC, Das JK, Salam RA, et al. Effective interventions to address maternal and child malnutrition: an update of the evidence. *Lancet Child Adolesc Health*. 2021;5(5):367-384. 10.1016/S2352-4642(20)30274-1 33691083

[6] Boerma T, Eozenou P, Evans D, et al. Monitoring progress towards universal health coverage at country and global levels. *PLoS Med*. 2014;11(9):e1001731. 10.1371/journal.pmed.1001731 25243899 PMC4171369

[7] Leyvraz M, Aaron GJ, Poonawala A, et al. Coverage of nutrition interventions intended for infants and young children varies greatly across programs: results from coverage surveys in 5 countries. *J Nutr*. 2017;147(5):995S-1003S. 10.3945/jn.116.245407 28404839 PMC5404212

[8] Menon P, Avula R, Pandey S, et al. Rethinking effective nutrition convergence an analysis of intervention co-coverage data. *Econ Pol Wkly*. 2019;54(24):18-21. https://www.epw.in/journal/2019/24/commentary/rethinking-effective-nutrition-convergence.html

[9] Bryce J, Arnold F, Blanc A, et al. Measuring coverage in MNCH: new findings, new strategies, and recommendations for action. *PLoS Med*. 2013;10(5):e1001423. 10.1371/journal.pmed.1001423 23667340 PMC3646206

[10] Nguyen PH, Singh N, Scott S, et al. Unequal coverage of nutrition and health interventions for women and children in seven countries. *Bull World Health Organ*. 2022;100(1):20-29. 10.2471/BLT.21.286650 35017754 PMC8722629

[11] Chakrabarti S, Raghunathan K, Alderman H, et al. India’s integrated child development services programme; equity and extent of coverage in 2006 and 2016. *Bull World Health Organ*. 2019;97(4):270-282. 10.2471/BLT.18.221135 30940984 PMC6438246

[12] Joe W, Alambusha R, Kulkarni B. Coverage of iron and folic acid supplementation in India: progress under the anemia Mukt Bharat strategy 2017-20. *Health Policy Plan*. 2022;37(5):597-606. 10.1093/heapol/czac015 35257147 PMC9113188

[13] Croft TN, Allen CK, Zachary BW. Guide to DHS Statistics. The Demographic and Health Surveys Program; 2023. Available from: https://www.DHSprogram.com

[14] Barros AJD, Victora CG. Measuring coverage in MNCH: determining and interpreting inequalities in coverage of maternal, newborn, and child health interventions. *PLoS Med*. 2013;10(5):e1001390. 10.1371/journal.pmed.1001390 23667332 PMC3646214

[15] Boerma JT, Bryce J, Kinfu Y, et al. Mind the gap: equity and trends in coverage of maternal, newborn, and child health services in 54 countdown countries. *Lancet*. 2008;371(9620):1259-1267. 10.1016/S0140-6736(08)60560-7 18406860

[16] Siyam A, Ir P, York D, et al. The burden of recording and reporting health data in primary health care facilities in five low- and lower-middle income countries. *BMC Health Serv Res*. 2021;21(suppl 1):691. 10.1186/s12913-021-06652-5 34511083 PMC8436492

[17] Erchick DJ, Subedi S, Verhulst A, et al. Quality of vital event data for infant mortality estimation in prospective, population-based studies: an analysis of secondary data from Asia, Africa, and Latin America. *Popul Health Metr*. 2023;21(1):10. 10.1186/s12963-023-00309-7 37507749 PMC10375772

[18] NITI Aayog . POSHAN Abhiyaan Progress Report. NITI Aayog, WCD Division; 2020. Available from: https://www.niti.gov.in/sites/default/files/2020-10/AbhiyaanMonitoringReport.pdf

[19] United Nations Department of Economic and Social Affairs . World Population Prospects 2022. United Nations; 2022.

[20] Menon P, Avula R, Sarswat E, et al. Tracking India’s progress on addressing malnutrition and enhancing the use of data to improve programs. *Pulkit Agarwal.* 2020. Available from:. https://www.ifpri.org/publication/tracking-indias-progress-addressing-malnutrition-and-enhancing-use-data-improve-programs

[21] IIPS . India: Standard DHS, 2019-21. The DHS Program. International Institute for Population Sciences; 2021.

[22] IIPS . India: Standard DHS, 2015-16. The DHS Program. International Institute for Population Sciences; 2017.

[23] Department of Economic and Social Affairs . World Population Prospects 2024: Methodology of the United Nations Population Estimates and Projections. United Nations; 2024. Available from: https://www.unpopulation.org

[24] IHME . GBD Compare. Institute for Health Metrics and Evaluation 2021. Accessed October 21, 2024. https://vizhub.healthdata.org/gbd-compare/

[25] Pachauri S . Priority strategies for India’s family planning programme. *Indian J Med Res*. 2014;140(suppl 1):S137-S146. PMID: 25673535. 25673535 PMC4345745

[26] Lassi ZS, Mansoor T, Salam RA, et al. Essential pre-pregnancy and pregnancy interventions for improved maternal, newborn and child health. *Reprod Health*. 2014;11(suppl 1):S2. 10.1186/1742-4755-11-S1-S2 PMC4145858 25178042

[27] UNIGME . Levels & Trends in Child Mortality: Report 2023. UNICEF; 2024.

[28] Masset E . SYNCMRATES: Stata Module to Compute Child Mortality Rates Using Synthetic Cohort Probabilities. Statistical Software Components; 2016.

[29] Yan T, Tourangeau R. Detecting underreporters of abortions and miscarriages in the National Study of Family Growth, 2011-2015. *PLoS One*. 2022;17(8):e0271288. 10.1371/journal.pone.0271288 35921280 PMC9348680

[30] CDC . Estimating the Number of Pregnant Women in a Geographic Area: A Reproductive Health Tool. National Center for Chronic Disease Prevention and Health Promotion; 2013. Available from: https://www.cdc.gov/reproductive-health/media/files/Pregnant-Population-Size-Estimator-397

[31] NSO . National Population and Housing Census 2021: National Report. Government of Nepal, Office of the Prime Minister and Council of Ministers, National Statistics Office; 2023.

[32] Ministry of Health and Population, New ERA, The DHS Program . Nepal Demographic and Health Survey 2022. The Demographic and Health Surveys Program; 2023. Available from: www.DHSprogram.com

[33] Nilsen K, Tejedor-Garavito N, Leasure DR, et al. A review of geospatial methods for population estimation and their use in constructing reproductive, maternal, newborn, child and adolescent health service indicators. *BMC Health Serv Res*. 2021;21(suppl 1):370. 10.1186/s12913-021-06370-y 34511089 PMC8436450

[34] Nambiar A, Choudhury DR, Agnihotri SB. Seasonal variations in childbirth: a perspective from the HMIS database (2017-2020). *Econ Pol Wkly*. 2022;57(17):38-45. https://www.epw.in/journal/2022/17/perspectives/seasonal-variations-childbirth.html

[35] Dorrington RE, Bradshaw D. Acknowledging uncertainty about maternal mortality estimates. *Bull World Health*. 2016;94(2):155-156. 10.2471/BLT.15.155036 PMC4750434 26908966

